# Mesenchymal stem cells in intrahepatic and extrahepatic biliary diseases: mechanisms and therapeutic applications

**DOI:** 10.3389/fcell.2026.1862138

**Published:** 2026-06-19

**Authors:** Meng-jiao Li, Wei Zhao, Cai-yang Li, Yan-ling Li, Shi-hui Ren, Jia-jia Li, Ling Qiu, Cheng-wei Yang, Fang-yi Fan, Hao Yao

**Affiliations:** 1 Department of Clinical Medicine, North Sichuan Medical College, Nanchong, Sichuan, China; 2 Department of Hematology, The General Hospital of Western Theater Command, Chengdu, Sichuan, China; 3 Department of Reproductive Medicine, The 924th Hospital of Joint Logistics Support Force of PLA, Guilin, Guangxi, China; 4 Institute of Basic Medicine, North Sichuan Medical College, Nanchong, Sichuan, China; 5 Department of Orthopaedics, The 940th Hospital of Joint Logistics Support Force of PLA, Lanzhou, Gansu, China; 6 College of Medicine, Southwest Jiaotong University, Chengdu, China; 7 Reproductive Medicine Center, The General Hospital of Western Theater Command, Chengdu, Sichuan, China; 8 Tissue Stress Injury and Functional Repair Key Laboratory of Sichuan Province, Chengdu, Sichuan, China

**Keywords:** biliary atresia (BA), biliary diseases, cholangiocarcinoma (CCA), liver fibrosis, mesenchymal stem cells, primary biliary cholangitis (PBC), primary sclerosing cholangitis (PSC)

## Abstract

Mesenchymal stem cells (MSCs) have emerged as promising therapeutic candidates for a wide range of intrahepatic and extrahepatic biliary diseases. However, their clinical application remains limited by an incomplete understanding of underlying mechanisms and heterogeneous therapeutic outcomes. This review provides a comprehensive overview of recent advances in MSC-based therapies for biliary system diseases, including primary biliary cholangitis, intrahepatic biliary fibrosis and cirrhosis, biliary atresia, primary sclerosing cholangitis, and cholangiocarcinoma. We summarize both preclinical and clinical evidence and focus on key therapeutic mechanisms, including immunomodulation, antifibrotic activity, regulation of bile acid metabolism, and protection of biliary epithelial cells.

## 1 Introduction

The bile ducts play a critical role in the transport of bile within the human body, with the bile duct epithelium performing essential secretion and absorption functions that maintain the stability of bile components—such as bile salts, cholesterol, and phospholipids—during the process of bile transport (Geng et al., 2025; Ooi et al., 2004). When the structure and function of the bile ducts become impaired, resulting in bile flow obstruction, a cascade of diseases related to the biliary system can ensue. Primary biliary cholangitis (PBC), a chronic cholestatic autoimmune liver disease, has been shown to be caused by long-term immune system attacks that lead to selective destruction of the small and medium intrahepatic bile ducts, resulting in intrahepatic bile stasis and inducing cholangiocyte hyperplasia (Hirschfield et al., 2017; Sarcognato et al., 2021). Biliary atresia (BA), a neonatal liver disease, is characterized by progressive obstruction and fibrosis of the extrahepatic bile ducts, accompanied by fibrosis and inflammation of the liver parenchyma (Wehrman et al., 2019). Primary sclerosing cholangitis (PSC), a progressive disease that leads to fibrosis and occlusion of the intrahepatic or extrahepatic bile ducts, is another significant condition affecting the biliary system (Poch et al., 2021). If left untreated, these biliary diseases can progress to liver fibrosis and cirrhosis. However, the current treatment options are limited, with many patients experiencing poor therapeutic outcomes, relapses, or other complications, thus underscoring the urgent need for the development of novel therapeutic approaches.

The International Society for Cellular Therapy (ISCT) has recommended the reclassification of cells commonly referred to as mesenchymal stem cells (MSCs), suggesting that they be termed “multipotent mesenchymal stromal cells (Renesme et al., 2025).” According to this terminology, “mesenchymal stem cells (MSCs)” should specifically refer to those subpopulations that meet clearly defined criteria for stem cell activity (Renesme et al., 2025). For both research and clinical application, the ISCT MSC Committee has advised the continued use of the MSC abbreviation, provided that the tissue source is carefully labeled, and emphasized the need for rigorous methods to validate their functionaliyt (Renesme et al., 2025). To this end, the ISCT MSC Committee has issued a consensus document outlining functional assays for MSCs, recommending the use of quantitative RNA analysis for specific genes, flow cytometry for cell surface markers, and proteomic analysis of the MSC secretome to confirm their functional capabilities (Renesme et al., 2025).

**FIGURE 1:**
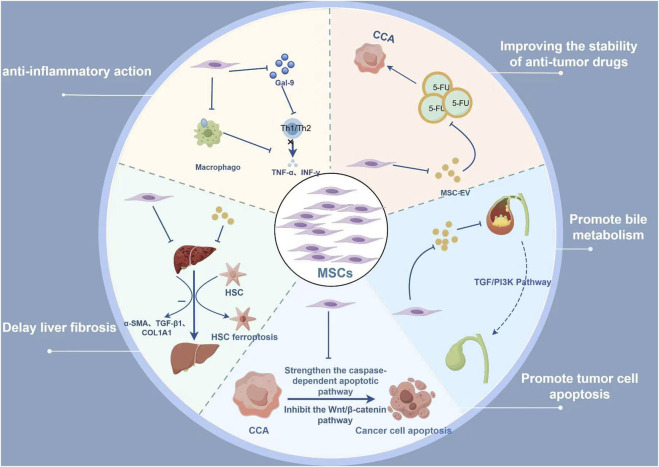
Mechanisms of Mesenchymal Stem Cells (MSCs) in Intrahepatic and Extrahepatic Biliary System Diseases (By FigDraw). MSCs exert multiple therapeutic effects on intrahepatic and extrahepatic biliary system diseases through various molecular mechanisms. (1) Anti-inflammatory action: MSCs secrete galectin-9 (Gal-9) to regulate the Th1/Th2 balance and suppress proinflammatory cytokines such as TNF-α and IFN-γ while modulating macrophage activation. (2) Delayed liver fibrosis: MSCs inhibit hepatic stellate cell (HSC) activation by downregulating the expression of fibrotic markers (α-SMA, TGF-β1, and COL1A1) and promoting HSC ferroptosis, thereby reducing fibrogenesis. (3) Promotion of bile metabolism: MSCs and their extracellular vesicles (MSC-EVs) regulate the TGF/PI3K signaling pathway to increase bile secretion and metabolic balance. (4) Improving antitumor drug stability: MSC-EVs enhance the therapeutic stability and delivery efficiency of 5-fluorouracil (5-FU) in cholangiocarcinoma (CCA). (5) Promotion of tumor cell apoptosis: MSCs inhibit the Wnt/β-catenin pathway and activate caspase-dependent apoptotic signaling, inducing the apoptosis of cholangiocarcinoma and other biliary-related cancer cells.

MSCs possess several properties that support their therapeutic application, including broad tissue distribution, relative ease of isolation and expansion, low immunogenicity, migratory capacity toward injury sites, multilineage differentiation potential, and immunomodulatory activity (Pittenger et al., 1999; Tavakoli et al., 2020; Beyth et al., 2005; Le Blanc et al., 2003; Caplan, 2016; Han et al., 2025; Abumaree et al., 2017; Zhang et al., 2017). These features have encouraged the investigation of MSC-based therapies in metabolic, cardiovascular, autoimmune, and inflammatory diseases (Zeinhom et al., 2024; Guo et al., 2020; Li et al., 2021). In hepatobiliary disorders, MSCs may contribute to tissue repair by regulating inflammatory responses, attenuating fibrosis, and supporting epithelial and metabolic homeostasis. In recent years, MSC-based therapies for intrahepatic and extrahepatic biliary diseases have become an active area of research. This review summarizes the clinical efficacy, safety, and underlying mechanisms of MSC-based therapies in primary biliary cholangitis, biliary atresia, primary sclerosing cholangitis, cholangiocarcinoma, and related biliary fibrosis and cirrhosis (Figure 1).

## 2 Literature search strategy

A comprehensive literature search was conducted using PubMed, Web of Science, Embase, and Scopus to identify relevant studies on mesenchymal stem cells (MSCs) in intrahepatic and extrahepatic biliary system diseases up to May 2025. The search strategy combined Medical Subject Headings (MeSH) and free-text terms, including “mesenchymal stem cells”, “mesenchymal stromal cells”, “biliary disease”, “primary biliary cholangitis”, “biliary atresia”, “primary sclerosing cholangitis”, “cholangiocarcinoma”, and “liver fibrosis”, with Boolean operators (AND, OR). Original research articles, clinical studies, and relevant reviews focusing on the therapeutic mechanisms and clinical applications of MSCs were included, while studies lacking direct relevance to hepatobiliary diseases were excluded. Reference lists of selected articles were also screened to identify additional relevant studies. Only articles published in English were considered, and studies were selected based on relevance and methodological quality.

## 3 MSCs in primary biliary cholangitis

### 3.1 Therapeutic mechanisms

PBC is a chronic cholestatic autoimmune liver disease characterized by the selective destruction of small- and medium-sized intrahepatic bile ducts due to persistent autoimmune attack (Hirschfield et al., 2017; Sarcognato et al., 2021). This results in intrahepatic cholestasis, cholangiocyte proliferation, and, as the disease progresses, progression to liver cirrhosis, hepatic failure, and associated complications (Sarcognato et al., 2021). Current therapeutic options remain limited, and for end-stage patients, liver transplantation remains the only effective intervention (Yang et al., 2022). However, the scarcity of donor organs and economic limitations necessitate the development of novel therapeutic strategies, among which mesenchymal stem cell therapy has shown significant promise.

Wang et al. established a PBC mouse model by administering polyinosinic–polycytidylic acid sodium (poly I:C) to C57BL/6 mice to evaluate the therapeutic effects of bone marrow–derived MSCs (BM-MSCs). Six weeks after MSC infusion, serum transaminase and autoantibody levels were markedly reduced (Wang et al., 2011). Histological analysis using hematoxylin–eosin staining demonstrated clear improvement in periductal mononuclear cell infiltration (Wang et al., 2011). Flow cytometric analysis further revealed that allogeneic BM-MSC transplantation significantly increased CD4+Foxp3+ regulatory T cells in peripheral blood and lymph nodes (Wang et al., 2011). Additionally, serum transforming growth factor β1 (TGF-β1) was elevated, interferon-γ (IFN-γ) was significantly reduced, while interleukin-10 (IL-10) remained unchanged (Wang et al., 2011). These results suggest that BM-MSC transplantation modulates systemic immune responses and facilitates hepatic inflammatory recovery in PBC patients (Wang et al., 2011).

Similarly, Fan et al. intravenously administered human umbilical cord–derived MSCs (UC-MSCs) to mice with autoimmune cholangitis induced by 2-octynoic acid–bovine serum albumin (2OA-BSA) conjugate (Fan et al., 2018). Compared with untreated controls, UC-MSC–treated mice exhibited significantly decreased serum alanine aminotransferase (ALT), aspartate aminotransferase (AST), alkaline phosphatase (ALP), and γ-glutamyl transpeptidase (GGT) levels, along with a marked reduction in anti–PDC-E2 autoantibodies. These findings indicate that UC-MSCs effectively suppress disease progression in experimental autoimmune cholangitis (Fan et al., 2018). Mechanistically, UC-MSCs modulated gene expression involved in immune and inflammatory pathways through the signal transducer and activator of transcription (STAT) and c-Jun N-terminal kinase (JNK) signaling pathways. Specifically, Galectin-9 (Gal-9) secreted by UC-MSCs inhibited CD4^+^ T-cell proliferation and suppressed Th1 and Th17 differentiation, thereby attenuating immune activation (Fan et al., 2018).

Cholangiocyte senescence is recognized as a key pathological process in PBC. Human placenta–derived MSC (hPMSC) exosomes have been shown to delay cholangiocyte aging (Chen et al., 2021). Chen et al. employed an advanced organoid culture system to establish oxidative stress–induced senescent cholangiocyte organoids, exploring whether hPMSC-derived exosomes confer protective effects (Chen et al., 2021). Treatment with hPMSC exosomes significantly delayed the senescence process of cholangiocyte organoids, associated with decreased expression of senescence-associated secretory phenotype (SASP) components and chemokines, including CCL2, CX3CL1, IL-6, TNF-α, CXCL1, and CXCL10 (Chen et al., 2021). These findings highlight the potential of MSC-derived exosomes in mitigating cholangiocyte senescence and inflammation, offering a novel therapeutic approach for PBC. However, these mechanistic findings are mainly derived from animal models and cholangiocyte organoids, and should therefore be distinguished from the clinical outcomes observed in patients with UDCA-resistant PBC (Wang et al., 2014; Wang et al., 2013).

### 3.2 Clinical applications

Clinical evidence has begun to demonstrate the therapeutic potential of MSCs in primary biliary cholangitis (PBC), particularly among patients with suboptimal response to ursodeoxycholic acid (UDCA) (Wang et al., 2014; Wang et al., 2013). In a prospective single-arm investigation, UC-MSCs were administered intravenously at a dose of 0.5 × 10^6^ cells/kg to seven patients who had shown poor response to UDCA (Wang et al., 2013). The regimen consisted of three infusions at 4-week intervals, with a total follow-up period of 48 weeks (Wang et al., 2013). Following MSC therapy, pronounced improvements in fatigue and pruritus were observed, accompanied by significant reductions in serum ALP and GGT levels at study completion compared with baseline values, indicating biochemical and symptomatic remission (Wang et al., 2013).

Using a similar therapeutic rationale, allogeneic BM-MSCT was employed in ten patients with UDCA-resistant primary biliary cirrhosis, representing the advanced stage of PBC (Wang et al., 2014). Throughout a 12-month observation period, no transplantation-related adverse events were reported, and patient-reported outcomes from the PBC-40 questionnaire confirmed substantial enhancement in quality of life (Wang et al., 2014). Post-treatment laboratory indices revealed significant decreases in ALT, AST, GGT, and IgM levels relative to baseline (Wang et al., 2014). Flow cytometric profiling of peripheral lymphocytes further demonstrated a decline in CD8^+^ T-cell proportions, whereas CD4^+^CD25+Foxp3+ T-cell frequency and serum IL-10 concentration were elevated (Wang et al., 2014).

Together, these findings substantiate the clinical feasibility, safety, and immunoregulatory efficacy of MSC-based therapy for PBC patients with incomplete or poor UDCA response, suggesting a valuable adjunctive role in managing cholestatic autoimmune liver disease (Wang et al., 2014; Wang et al., 2013).

## 4 MSCs in intrahepatic bile duct fibrosis and cirrhosis

### 4.1 Therapeutic mechanisms

Cirrhosis, a major complication of chronic liver disease, represents the advanced stage of hepatic fibrosis and is characterized by hepatocellular injury and irreversible scarring of the liver parenchyma (Yang et al., 2023). Endogenous mesenchymal stem cells (MSCs) are known to play a crucial role in the onset and progression of hepatic fibrosis (Yang et al., 2023). A growing body of evidence suggests that MSCs contribute to both the formation and regulation of myofibroblast (MF) populations, the key effector cells driving fibrogenesis (Yang et al., 2023; Parola and Pinzani, 2019; Matsuda and Seki, 2020). Indeed, MSCs themselves can serve as one of the cellular sources of hepatic MFs and can modulate the myofibroblastic activation of hepatic stellate cells (HSCs) (Yang et al., 2023; Parola and Pinzani, 2019; Matsuda and Seki, 2020).

In addition to their structural contributions, MSCs participate in multiple regulatory processes, including immune modulation during liver injury and repair, the initiation and resolution of acute inflammation, and the maintenance of chronic inflammatory states, thereby influencing the overall course of hepatic fibrogenesis (Yang et al., 2023). Currently, MSC-based therapy has emerged as one of the most actively explored and promising strategies for treating hepatic fibrosis and cirrhosis. Several comprehensive reviews have delineated the therapeutic mechanisms of MSCs in this context (Yang et al., 2023; Li et al., 2025; Watanabe et al., 2021), which can be summarized as follows:

1. Differentiation into functional hepatocytes;
2. Attenuation of inflammatory responses and protection of hepatocytes from injury;
3. Inhibition of HSC activation;
4. Modulation of the hepatic inflammatory microenvironment;
5. MSC-derived extracellular vesicles (MSC-EVs) alleviate hepatocellular injury by regulating macrophage polarization, autophagy, the secretion of proinflammatory cytokines such as TGF-β and IL-6, and oxidative stress.

Although significant progress has been achieved, many underlying mechanisms remain to be elucidated. Emerging studies continue to reveal novel insights. Recent findings indicate that MSCs may exert antifibrotic effects by inhibiting intrahepatic B-cell proliferation and cytokine production through exosome-mediated modulation of the mitogen-activated protein kinase (MAPK) and nuclear factor kappa B (NF-κB) pathways (Feng et al., 2025). This observation provides a new perspective on the immunomodulatory actions of MSCs in hepatic fibrosis. Furthermore, Wang et al. reported that MSC-derived exosomes (MSC-Exos) deliver miR-499a-5p, which interacts with the transcription factor ETS1 to suppress glutathione peroxidase 4 (GPX4) expression, thereby promoting ferroptosis in HSCs and reducing their profibrotic activity (Wang et al., 2025). Overexpression of ETS1 in HSCs reversed miR-499a-5p–induced ferroptosis and restored HSC activation, offering a mechanistic explanation for the antifibrotic potential of human umbilical cord–derived MSCs (hUC-MSCs) (Wang et al., 2025). Compared with these mechanism-oriented preclinical studies, clinical investigations in cirrhosis have mainly evaluated patient-level outcomes, including liver function, survival, and treatment-related adverse events (Shi et al., 2021; Li et al., 2023; Wang et al., 2023).

### 4.2 Clinical applications

The therapeutic efficacy and safety of MSC therapy in liver cirrhosis have been substantiated by multiple clinical studies. Among them, the largest to date was conducted by Shi et al., who performed a prospective, open-label, randomized controlled trial enrolling 219 patients with hepatitis B virus (HBV)–related decompensated liver cirrhosis (DLC) (Shi et al., 2021). Participants were assigned to either a control group (n = 111) or a treatment group receiving UC-MSC infusions (n = 108), with a follow-up period extending up to 7 years. The overall survival rate was significantly higher in the MSC-treated group, although no difference was observed in hepatocellular carcinoma–free survival between groups (Shi et al., 2021). UC-MSC therapy markedly improved hepatic function, as reflected by enhanced serum albumin, prothrombin activity, cholinesterase, and total bilirubin levels during the 48-week follow-up. Importantly, no significant adverse reactions or treatment-related complications were reported (Shi et al., 2021).

In a more recent investigation involving 201 participants, 36 patients received human umbilical cord blood–derived MSC transplantation (HUCB-MSCT), while 165 served as non-transplanted controls (Li et al., 2023). The three- and five-year survival rates in the transplanted cohort (83.3% and 63.9%, respectively) were notably higher than those in the control group (61.8% and 43.6%), with median overall survival times of 92.5 and 50.8 months, respectively (Li et al., 2023).

Complementing these findings, a meta-analysis incorporating 13 clinical studies and 854 patients with liver cirrhosis or acute liver failure confirmed that MSC therapy significantly improved hepatic parameters—including Model for End-Stage Liver Disease (MELD) scores, total bilirubin (TB), and albumin (ALB) levels—compared with conventional therapy (Wang et al., 2023). Moreover, overall survival was significantly enhanced among patients with cirrhosis and acute-on-chronic liver failure (ACLF), with no severe adverse events or safety concerns reported following MSC treatment (Wang et al., 2023).

Collectively, these clinical data underscore the long-term safety, tolerability, and efficacy of MSC-based therapy as a promising adjunctive treatment for hepatic fibrosis and cirrhosis, offering renewed hope for patients with advanced liver disease (Shi et al., 2021; Li et al., 2023; Wang et al., 2023).

## 5 MSCs in biliary atresia

### 5.1 Therapeutic mechanisms

BA is a severe neonatal liver and biliary disease that ultimately leads to liver failure (Zagory et al., 2015). Current treatment options primarily include Kasai portoenterostomy; however, the prognosis for most patients remains poor, and the final treatment often relies on orthotopic liver transplantation (Zagory et al., 2015). Consequently, the development of novel therapies for BA is urgently needed. Human exfoliated deciduous teeth (SHED) express mesenchymal stem cell (MSC) properties, which make them a potential source of stem cells for therapeutic applications (Sonoda et al., 2021). Sonoda et al. investigated the *in vitro* stem cell properties and biliary potential of SHED derived from healthy donors (Cont-SHED) and BA patients (BA-SHED) (Sonoda et al., 2021). Their results demonstrated that SHED stem cells possess the ability to directly transdifferentiate into cholangiocytes and hepatocytes within liver tissue. Moreover, SHED can induce regeneration of cholangiocytes and hepatocytes in a CCl_4_-induced liver fibrosis mouse model, significantly improving bile drainage and liver function in these fibrosis models (Sonoda et al., 2021).

Further studies by Lei et al. employed a mouse model of BA induced by Rous Sarcoma Virus (RSV) and treated the animals with bone marrow–derived MSCs (BMMSCs) via intraperitoneal injection (Lei et al., 2018). Following BMMSC therapy, the levels of liver enzymes and bilirubin metabolism, oxidative stress markers such as malondialdehyde (MDA), fibrosis markers, and liver fibrosis-related proteins (including collagen IV, laminin, α-SMA, COL1A1, TGF-β1, and TNF-α) were significantly reduced (Lei et al., 2018). Furthermore, the levels of superoxide dismutase and glutathione peroxidase were elevated in the liver tissues (Lei et al., 2018). Histological examination of liver sections from the BA group revealed extensive diffuse and progressive tissue alterations, with substantial mononuclear cell infiltration and pronounced fibrosis (Lei et al., 2018). In contrast, the BMMSC-treated group showed a marked reduction in these histopathological changes, suggesting that BMMSCs can effectively inhibit the progression of fibrosis in this mouse model of BA (Lei et al., 2018).

### 5.2 Clinical applications

The clinical application of MSCs in BA has been explored in several studies. Degtyareva et al. used multipotent mesenchymal stromal cells to treat two patients with post-Kasai hepatic dysfunction and portal hypertension, both of whom did not respond adequately to standard therapy (Degtyareva et al., 2022). Both patients were candidates for liver transplantation, but during MSC treatment, significant relief of the inflammatory process and functional recovery of the liver were achieved (Degtyareva et al., 2022). During follow-up, no recurrence of cholangitis was observed, and liver function was preserved (Degtyareva et al., 2022). These results indicate the feasibility of using MSC therapy to manage complications in children following Kasai surgery for BA (Degtyareva et al., 2022).

## 6 MSCs in primary sclerosing cholangitis

### 6.1 Therapeutic mechanisms

Primary sclerosing cholangitis (PSC) is a progressive fibrosing disease of intrahepatic or extrahepatic bile ducts, typically leading to liver cirrhosis, liver failure, and death (Poch et al., 2021). Effective treatment options for PSC remain limited, and it often progresses to liver transplantation. Sonoda et al. conducted a study in an MDR2 knockout mouse model, demonstrating that mesenchymal stem cell (MSC)-derived extracellular vesicles (EVs) can prevent the accumulation of hepatotoxic CD8^+^ T cells in the liver and reduce neutrophil infiltration (Angioni et al., 2020). This study highlights the potential of MSC-derived EVs to inhibit PSC progression by reducing periductal fibrosis and inflammation (Angioni et al., 2020).

In another study, Ryo Sugiura et al. explored the effects of human amniotic mesenchymal stem cells (hAMSCs) and conditioned medium (CM) derived from hAMSCs in rats with sclerosing cholangitis (Sugiura et al., 2018). The results showed that both hAMSC transplantation and CM administration significantly reduced the mRNA levels of matrix metalloproteinase-9 (MMP-9), and tended to lower the mRNA levels of α-smooth muscle actin (α-SMA), transforming growth factor-beta (TGF-β), type I collagen, MMP-2, and tissue inhibitor of metalloproteinase-1 (TIMP-1) (Sugiura et al., 2018). Moreover, hAMSCs and CM inhibited ANIT-induced cholangiocyte proliferation, suggesting their potential to reduce periductal fibrosis by suppressing inflammation and subsequent cholangiocyte hyperplasia (Sugiura et al., 2018).

The T-helper 17 (Th17) cell subset plays a pivotal role in the fibrosis process of PSC (Poch et al., 2021; Kunzmann et al., 2020; Chen et al., 2024). Chen et al. developed MSC-derived exosomes (ExoMSC) and further investigated their antifibrotic effects and detailed mechanisms in PSC using Mdr2−/− mice and multicellular organoids derived from PSC patients (Chen et al., 2024). ExoMSC treatment improved liver fibrosis in Mdr2−/− mice, and RNA sequencing analysis revealed a significant reduction in collagen deposition in the periductal area, as well as inhibition of Th17 differentiation (Chen et al., 2024). Additionally, the percentage of CD4^+^ IL-17A+ T cells was reduced in ExoMSC-treated Mdr2−/− mice (Chen et al., 2024). Further research confirmed that ExoMSC alleviates liver fibrosis in PSC by downregulating IκBζ expression, which inhibits Th17 differentiation. ExoMSC also improves the Th17-induced microenvironment by modulating the PERK/CHOP signaling pathway and restoring the hypersecretory phenotype of cholangiocytes, as well as reducing the interaction between hepatic stellate cells (HSCs) and cholangiocytes (Chen et al., 2024). These findings suggest that ExoMSC could be a potential therapeutic approach for PSC-related liver fibrosis and other Th17-associated diseases.

In a study by Yao et al., liver tissue samples from PSC patients were collected to construct intrahepatic cholangiocyte organoids, and several bile duct obstruction disease models were developed (Yao et al., 2023). The therapeutic effects of human placenta–derived MSCs (hP-MSCs) on Mdr2−/− mice were primarily mediated by *in vivo* secretion of insulin-like growth factor 1 (IGF-1) and downregulation of CXCL1/2, which target TGR5 on cholangiocytes (Yao et al., 2023). Ultimately, MSC treatment improved the inflammatory phenotype and inhibited organoid PSC liver proliferation through the TGR5/PI3K/ERK and TGR5/Pellino3/NF-κB signaling pathways (Yao et al., 2023). Furthermore, Yu et al. demonstrated that the majority of bile acids were significantly elevated in the model group but decreased following hP-MSC treatment (Yu et al., 2024). These results revealed that MSCs regulate key bile acid transporters and enzymes involved in bile acid synthesis, thereby modulating the entire bile acid metabolism network and alleviating the systemic adverse effects of cholestasis (Yu et al., 2024). The key mechanisms of MSCs across different biliary diseases are summarized in Table 1. Nevertheless, most current evidence for PSC remains preclinical, relying on Mdr2−/− mice, ANIT-induced models, and patient-derived organoids, whereas clinical validation is still limited to very early experience (Lightner et al., 2023).

**TABLE 1:** Summary of mechanisms of MSCs in intrahepatic and extrahepatic biliary tract diseases MSCs.

Disease type	Cell type	Experimental model	Main mechanisms	References
PBC	BM-MSCs	Poly I:C-induced mouse	Increased Treg cells, decreased IFN-γ, regulation of bile duct inflammation	Wang et al. (2011)
PBC	UC-MSCs	2OA-BSA-induced mouse	Inhibition of Th1/Th17 differentiation via Gal-9, regulation of STAT/JNK pathway, attenuated immune activation	Fan et al. (2018)
PBC	hPMSC-Exos	Cholangioid organoid	Decreased SASP and chemokine expression, delayed cholangiocyte senescence	Chen et al. (2021)
LF/Cirrhosis	MSCs	CCl_4_-induced mouse	Mediation of MAPK/NF-κB pathway, Inhibition of intrahepatic B-cell proliferation and cytokine production through modulation of the MAPK/NF-κB pathway	Feng et al. (2025)
LF/Cirrhosis	MSCs-Exos	CCl_4_-induced mouse	Delivery of miR-499a-5p, co-inhibition of GPX4 expression with ETS1, promotion of HSC ferroptosis, delayed fibrosis	Wang et al. (2025)
BA	SHED	CCl_4_-induced mouse	Induction of cholangiocyte and stem cell regeneration, improved bile drainage and liver function	Sonoda et al. (2021)
BA	BM-MSCs	RRV-induced mouse	Decreased oxidative stress markers, fibrosis markers, and related proteins; attenuation of liver fibrosis	Lei et al. (2018)
PSC	hAMSCs and hAMSC-CM	ANIT-induced rat	Decreased mRNA levels of MMP-9, MMP-2, TGF-β, TIMP-1; inhibition of ANIT-induced cholangiocyte proliferation, reduced fibrosis	Sugiura et al. (2018)
PSC	MSCs-Exos	Mdr2^−/−^mouse	Inhibition of Th17 cell differentiation, regulation of PERK/CHOP pathway; reduction of HSC–cholangiocyte interaction	Chen et al. (2024)
PSC	hP-MSCs	Cholangiocyte organoid, Mdr2^−/−^ mouse	Upregulation of TGR5 and regulation of the TGR5/PI3K/ERK and TGR5/PI3K/ERK and TGR5/Pellino3/NF-κB pathways; inhibition of cholangiocyte inflammatory activation and proliferation	Yao et al. (2023)
PSC	hP-MSCs	Mdr2^−/−^mouse	Regulation of bile acid synthesis-related transporters and enzymes; remodeling of the bile acid metabolic network	Yu et al. (2024)
CCA	CH-CM	CCA cell lines	Inhibition of JAK2/STAT3 signaling pathway, downregulation of BCL-2, upregulation of BAX	Jantalika et al. (2022)
CCA	UC-MSCs-Exos	CCA cell lines	Downregulation of CHEK1, promotion of tumor cell apoptosis, inhibition of EMT	Li and Wang (2022)
CCA	BM-MSCs-Exos	CCA cell lines	Inhibition of VEGFA, VEGFR2 and Wnt/β-catenin pathway, reduced tumor cell viability	Song et al. (2025)
CCA	Hu-MSC-Exos	CCA cell lines	Regulation of miR-620, enhanced APAF1 expression, inhibition of tumor cell proliferation and metastasis	Yuan et al. (2024)
CCA	5-Fu-Exos	CCA cell lines	Maintenance of 5-Fu stability, improved drug efficacy	Chen et al. (2022)

### 6.2 Clinical applications

The clinical use of MSCs in PSC has been evaluated in several studies. Lightner et al. treated a patient with progressive PSC by directly injecting 1.5 × 10^8^ GMP-derived MSCs into the common bile duct (Lightner et al., 2023). After *in vitro* evaluation, no adverse events or serious adverse events related to MSC treatment were observed (Lightner et al., 2023). Notably, no disease progression was detected within 6 months following MSC injection, although the study involved only a single patient and did not show significant therapeutic effects (Lightner et al., 2023). Nonetheless, the safety of MSC treatment was confirmed, highlighting the need for further clinical trials to validate the clinical efficacy of MSCs in PSC patients. A summary of current clinical applications of MSCs in biliary diseases is presented in Table 2.

**TABLE 2:** Summary of clinical applications of MSCs in intrahepatic and extrahepatic biliary tract diseases.

Disease type	Cell type	Number of patients	Administration route	Efficacy	Safety	References
PBC	UC-MSCs	7	Intravenous injection	Improves fatigue/pruritus, reduces ALP/GGT	Favorable	Wang et al. (2013)
PBC	BM-MSCs	10	Intravenous injection	Improves quality of life, reduces ALT/AST/GGT/IgM	Favorable	Wang et al. (2014)
LF/Cirrhosis	UC-MSCs	219	Intravenous injection	Increases survival rate, improves liver function	Favorable	Shi et al. (2021)
LF/Cirrhosis	UC-MSCs	201	Intravenous injection	Extends survival time, improves 3-/5-year survival	Favorable	Li et al. (2023)
LF/Cirrhosis	MSCs	854 (meta-analysis)	Intravenous injection	Improves liver function, lowers MELD score, prolongs survival	Favorable	Wang et al. (2023)
BA	MSCs	2	Intravenous injection	Resolves inflammation, maintains normal liver function long-term	Favorable	Degtyareva et al. (2022)
PSC	MSCs	1	Common bile duct injection	No disease progression at 6 months	Favorable	Lightner et al. (2023)

## 7 MSCs in cholangiocarcinoma (CCA)

Previous studies have shown that mesenchymal stem cells (MSCs) can inhibit tumor cell apoptosis, stimulate metastasis, and induce chemotherapy resistance by releasing various cytokines (Gan et al., 2021; Sueca-Comes et al., 2024; Zhong et al., 2017). However, a growing body of evidence now suggests that MSCs possess potent anticancer effects on cholangiocarcinoma (CCA) cells. To explore this, Jantalika et al. isolated MSCs from chorionic villus tissue (CH-CM) and examined their effects on three CCA cell lines—KKU100, KKU213A, and KKU213B—using MTT assays, annexin V/PI analysis, and JC-1 staining (Jantalika et al., 2022). The results showed that CH-CM inhibited CCA cell proliferation and promoted apoptosis, with the apoptotic effect being linked to mitochondrial-mediated caspase activation (Jantalika et al., 2022). Western blot analysis revealed that CH-CM inhibited JAK2/STAT3 signaling, downregulated BCL-2 expression, and increased the expression of BAX in CCA cells, thereby exerting antitumor effects (Jantalika et al., 2022).

Further research by Li et al. focused on miR-15a-5p, which has been implicated in the metastasis and invasiveness of malignant tumors and identified as a tumor suppressor in human malignancies (Li and Wang, 2022). They used exosomes derived from umbilical cord MSCs (UC-MSCs) loaded with miR-15a-5p to investigate its inhibitory effects on epithelial-mesenchymal transition (EMT) and metastasis in CCA (Li and Wang, 2022). The results demonstrated that miR-15a-5p targeted CHEK1 and downregulated its expression, with high CHEK1 expression in CCA being significantly associated with tumor biological characteristics such as tumor size, lymph node metastasis, cancer cell proliferation, invasion, migration, and EMT (Li and Wang, 2022). Additionally, UC-MSC-exosomes were found to suppress malignant progression, with the inhibition of apoptosis in CCA cells being mediated through CHEK1 (Li and Wang, 2022). Upon silencing miR-15a-5p, UC-MSC-exosomes exhibited opposing effects in CCA, suggesting that overexpression of miR-15a-5p in UC-MSC-exosomes promotes apoptosis and inhibits the malignant features of CCA cells and EMT by downregulating CHEK1 (Li and Wang, 2022).

A recent study revealed that endogenous TIMP2 encapsulated in BMSC-derived exosomes (BMSC-Exo/CS) inhibits key angiogenesis factors (VEGFA and VEGFR2) and the Wnt/β-catenin pathway (including β-catenin and c-Myc), thereby reducing CCA cell viabilit (Song et al., 2025). Notably, these inhibitory effects were reversed by the Wnt signaling agonist BML-28. Similarly, Yuan et al. discovered that Hu-MSC-derived exosomes containing circ_0037104 suppressed CCA cell proliferation and metastasis by regulating miR-620 and enhancing APAF1 expression (Yuan et al., 2024).

5-fluorouracil (5-Fu) is a widely used and cost-effective chemotherapy drug for CCA treatment, but its short half-life (10–20 min) and variability in absorption, distribution, metabolism, and pharmacokinetics limit its therapeutic effectiveness (Chen et al., 2022). Given the potential of MSC-derived exosomes as drug delivery vehicles for proteins, nucleic acids, and therapeutic molecules, Chen et al. loaded 5-Fu into exosomes (5-Fu-Exos) using ultrasound treatment and incubation methods (Chen et al., 2022). The results indicated that 5-Fu-Exos were more effective than free 5-Fu in inhibiting CCA cell viability, suggesting that MSC-derived exosomes could serve as a novel targeted drug delivery system for CCA therapy (Chen et al., 2022).

However, to date, no clinical trials involving MSCs or their derivatives in the treatment of CCA have been reported. Depending on microenvironmental conditions, MSCs can exhibit either tumor-suppressive or tumor-promoting effects, which may influence cholangiocarcinoma progression and therapeutic outcomes. Therefore, MSC-based strategies in CCA require careful preclinical evaluation before clinical translation (Gan et al., 2021; Sueca-Comes et al., 2024; Zhong et al., 2017; Jantalika et al., 2022; Li and Wang, 2022; Song et al., 2025; Yuan et al., 2024).

## 8 Discussion

In recent years, the incidence and mortality of diseases related to both intrahepatic and extrahepatic biliary systems seem to have been controlled with the current available pharmacological and surgical treatments. However, some patients still exhibit poor responses to these therapies, underscoring the urgent need for new treatment options. Mesenchymal stem cells (MSCs) have emerged as a popular topic in the treatment of various systemic diseases. The International Society for Cellular Therapy (ISCT) has recommended renaming the currently described “mesenchymal stem cells” (MSC) to “multipotent mesenchymal stromal cells (Renesme et al., 2025).” The term “mesenchymal stem cells” should specifically refer to those subpopulations that meet well-defined criteria for stem cell activity (Renesme et al., 2025). For research and clinical translational applications, the ISCT MSC committee suggests continuing to use the MSC abbreviation, but with explicit labeling of tissue sources—for example, bone marrow-derived MSCs (BM-MSCs), adipose-derived MSCs (AD-MSCs), and umbilical cord-derived MSCs (UC-MSCs) (Renesme et al., 2025). Moreover, rigorous methods should be employed to validate the functional characteristics of MSCs. The ISCT MSC committee has published a consensus document on functional assays, recommending three approaches to verify their functionality: quantitative RNA analysis of selected genes, flow cytometry for cell surface markers, and proteomic analysis of the MSC secretome (Renesme et al., 2025).

**FIGURE 2:**
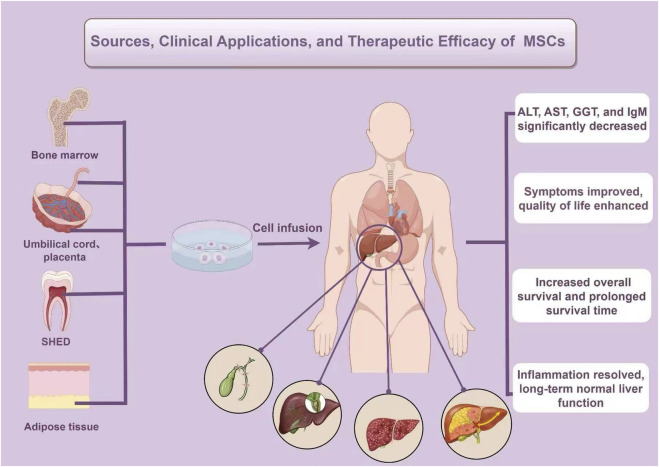
MSC sources and therapeutic effects in biliary diseases (By FigDraw). SCs derived from bone marrow, umbilical cord/placenta, adipose tissue, and stem cells from human exfoliated deciduous teeth (SHED) can be expanded *in vitro* and administered systemically, predominantly via intravenous infusion. Across a spectrum of intrahepatic and extrahepatic biliary diseases, including primary biliary cholangitis, liver fibrosis/cirrhosis, biliary atresia, and primary sclerosing cholangitis, MSC therapy is associated with improved liver biochemistry (reduced ALT, AST, GGT, and IgM), amelioration of clinical symptoms, enhanced quality of life, and prolonged survival. In addition, MSCs promote resolution of inflammation and support long-term hepatic functional recovery, underscoring their therapeutic potential in biliary system disorders.

In the preclinical studies we reviewed, all the MSCs used were shown to have relevant functional properties. Although their therapeutic mechanisms have not yet been fully elucidated, we summarize the existing mechanisms on the basis of current findings. MSCs modulate inflammation by suppressing inflammatory cytokine production and reshaping the inflammatory microenvironment, thereby alleviating tissue injury (Wang et al., 2011; Fan et al., 2018; Yang et al., 2023; Angioni et al., 2020; Sugiura et al., 2018; Yao et al., 2023) (Figure 1). Across different biliary diseases, the dominant mechanisms of MSCs appear to vary according to disease context. In PBC and PSC, immunomodulation and cholangiocyte protection are more prominent, whereas in biliary fibrosis and cirrhosis, antifibrotic effects involving HSC regulation, ferroptosis, and profibrotic signaling are more central (Wang et al., 2011; Fan et al., 2018; Yang et al., 2023; Wang et al., 2025; Angioni et al., 2020; Sugiura et al., 2018; Kunzmann et al., 2020; Chen et al., 2024; Yao et al., 2023; Yu et al., 2024). In PSC, additional modulation of bile acid metabolism and cholangiocyte–immune interactions may further contribute to disease-specific effects (Yao et al., 2023; Yu et al., 2024). In cholangiocarcinoma, the antitumor effects of MSCs or MSC-derived extracellular vesicles remain largely preclinical, and their application requires cautious interpretation (Jantalika et al., 2022; Li and Wang, 2022; Song et al., 2025; Yuan et al., 2024; Chen et al., 2022). Additionally, under certain induction conditions, MSCs can differentiate into target cells or tissues (Sonoda et al., 2021). However, the specific inducers and conditions still require further investigation. MSCs also play an important role in regulating bile acid metabolism, which contributes to functional recovery and delays damage to target organs and cells (Sonoda et al., 2021; Lei et al., 2018; Yu et al., 2024). In liver fibrosis patients, MSCs can inhibit hepatic stellate cell (HSC) activation, thus delaying the progression of liver fibrosis (Yang et al., 2023). In cholangiocarcinoma, MSCs are critical for inhibiting tumor cell proliferation and metastasis, as well as inducing tumor cell apoptosis (Jantalika et al., 2022). The sources, clinical applications, and overall therapeutic efficacy of MSCs are summarized in Figure 2.

MSC-derived extracellular vesicles (EVs) have also demonstrated significant potential in the treatment of intrahepatic and extrahepatic biliary diseases. Like MSCs, EVs modulate inflammatory responses and exert therapeutic effects (Li et al., 2025; Angioni et al., 2020). Furthermore, owing to their ability to transport proteins, nucleic acids, and therapeutic molecules and to deliver bioactive substances through multiple pathways and target sites, MSC-derived EVs improve the pharmacokinetic properties of chemotherapy drugs, including their short half-life, absorption, distribution, metabolism, and pharmacokinetics (Chen et al., 2022). Additionally, MSC-derived EVs play an important role in delaying cholangiocyte senescence (Chen et al., 2021) (Figure 1).

This review also summarizes the clinical trials involving the use of MSCs in the treatment of PBC, intrahepatic biliary fibrosis and cirrhosis, BA, PSC, and cholangiocarcinoma, where their efficacy and safety have been confirmed. Shi et al. conducted a large-sample, long-term follow-up randomized controlled trial involving 219 patients with HBV-related cirrhosis (Shi et al., 2021). The control group (n = 111) and UC-MSC treatment group (n = 108) were followed for up to 7 years. The survival rate in the treatment group was significantly greater than that in the control group, and liver function improved significantly, with the serum ALB, prothrombin activity, cholinesterase, and total bilirubin levels improving markedly (Shi et al., 2021). Importantly, no significant side effects or treatment-related complications were observed in the treatment group (Shi et al., 2021). A more recent study involving 201 participants, including 36 patients who received human umbilical cord blood-derived MSC transplantation (HUCB-MSCT) and 165 controls, reported 3-year and 5-year survival rates of 83.3% vs. 61.8% and 63.9% vs. 43.6%, respectively, with median overall survival (OS) times of 92.5 and 50.8 months, further supporting the efficacy of MSC treatment (Li et al., 2023). Degtyareva et al. used MSCs to treat two patients with liver cell dysfunction and portal hypertension after Kasai surgery, both of whom had not responded adequately to standard therapy (Degtyareva et al., 2022). The inflammation was controlled, liver function was restored, and no recurrence of cholangitis was observed during the follow-up, indicating the feasibility of MSC therapy for post-Kasai complications in pediatric BA patients (Degtyareva et al., 2022). Wang et al. used allogeneic bone marrow-derived MSC transplantation (BM-MSCT) to treat 10 PBC patients resistant to UDCA (Wang et al., 2014). Following treatment, the serum ALT, AST, GGT, and IgM levels decrease significantly, while the percentage of CD8^+^ T cells in peripheral lymphocyte subsets decreases, and the percentage of CD4^+^CD25^+^Foxp3^+^ T cells and the serum IL-10 level increase (Wang et al., 2014). Patients also reported an improvement in their quality of life (Wang et al., 2014). Although clinical experience with MSCs is still limited, the studies published to date have confirmed their efficacy and safety.

However, the current evidence should be interpreted with several methodological and biological constraints in mind. MSCs isolated from bone marrow, umbilical cord, placenta, adipose tissue, or exfoliated deciduous teeth do not necessarily represent interchangeable therapeutic products. Differences in proliferative behavior, immune-regulatory activity, secreted factors, lineage tendency, and tissue-targeting ability may influence their performance in different biliary disease settings (Renesme et al., 2025; Tavakoli et al., 2020; Abumaree et al., 2017; Zhang et al., 2017; Sonoda et al., 2021; Chen et al., 2022). In addition to tissue origin, product-related variables may also affect treatment consistency. Donor characteristics, culture medium, oxygen tension, passage number, cryopreservation procedures, release testing, and potency assays are not uniform across studies, making it difficult to directly compare therapeutic outcomes or define an optimal MSC product. More rigorous source annotation, functional characterization, and batch-release standards are therefore needed in future studies.

The safety profile of MSC-based therapy also requires a disease-specific assessment rather than a general assumption of safety. This is especially important for cholangiocarcinoma. Existing studies suggest that MSCs or MSC-derived extracellular vesicles can interfere with cholangiocarcinoma growth, angiogenesis, metastasis, and drug-resistance-related signaling (Jantalika et al., 2022; Li and Wang, 2022; Song et al., 2025; Yuan et al., 2024). However, opposite effects have also been reported, including enhanced chemoresistance, metastatic growth, and tumor progression under inflammatory or tumor-associated microenvironmental conditions (Gan et al., 2021; Sueca-Comes et al., 2024; Zhong et al., 2017). Therefore, MSC-based strategies for cholangiocarcinoma or premalignant biliary lesions should be considered cautiously. Although available clinical studies have not reported major short- or mid-term safety signals, the evidence remains limited by small cohorts, heterogeneous disease backgrounds, and inconsistent follow-up durations (Wang et al., 2014; Wang et al., 2013; Shi et al., 2021; Li et al., 2023; Wang et al., 2023; Degtyareva et al., 2022; Lightner et al., 2023). Future investigations should incorporate long-term surveillance, predefined safety endpoints, standardized cell-product evaluation, and adequately powered randomized designs to clarify both benefit and risk.

## 9 Conclusion

MSCs have emerged as promising therapeutic candidates for a broad spectrum of intrahepatic and extrahepatic biliary system diseases. Accumulating preclinical and clinical evidence indicates that MSCs exert therapeutic effects primarily through immunomodulation, attenuation of fibrogenesis, regulation of bile acid metabolism, and protection of biliary epithelial cells. In addition to cell-based therapies, MSC-derived extracellular vesicles represent a rapidly evolving cell-free approach with advantages in safety, stability, and targeted delivery.

Clinical studies to date suggest that MSC-based therapies are generally safe and may provide clinical benefits in conditions such as primary biliary cholangitis, biliary atresia, and liver cirrhosis. However, high-quality evidence remains limited, particularly for primary sclerosing cholangitis and cholangiocarcinoma, where clinical translation is still in its early stages.

Despite encouraging progress, several critical challenges must be addressed before widespread clinical implementation can be achieved. These include optimization of MSC sources, standardization of cell and extracellular vesicle preparation protocols, determination of appropriate dosing and administration routes, and comprehensive evaluation of long-term safety and efficacy. Moreover, in malignancies such as cholangiocarcinoma, the dual potential of MSCs to either suppress or promote tumor growth depending on microenvironmental cues underscores the necessity for rigorous preclinical assessment prior to clinical application.

Future research should prioritize well-designed, large-scale randomized controlled trials and the development of standardized therapeutic frameworks. Such efforts will be essential to fully elucidate the clinical value of MSC-based therapies and to facilitate their translation into effective treatment strategies for biliary system diseases.
